# Waterborne outbreak of tularemia associated with crayfish fishing.

**DOI:** 10.3201/eid0707.010740

**Published:** 2001

**Authors:** P Anda, J Segura del Pozo, J M Díaz García, R Escudero, F J García Peña, M C López Velasco, R E Sellek, M R Jiménez Chillarón, L P Sánchez Serrano, J F Martínez Navarro

**Affiliations:** Instituto de Salud Carlos III, Majadahonda, Madrid, Spain. panda@isciii.es

## Abstract

In 1997, an outbreak of human tularemia associated with hare-hunting in central Spain affected 585 patients. We describe the identification of Francisella tularensis biovar palaearctica in a second outbreak of ulceroglandular tularemia associated with crayfish (Procambarus clarkii) fishing in a contaminated freshwater stream distant from the hare-associated outbreak. The second outbreak occurred 1 year after the first.


### Waterborne Outbreak of Tularemia Associated with Crayfish Fishing

**Pedro Anda,* Javier Segura del Pozo,*† José María Díaz García,‡ Raquel Escudero,* F. Javier García Peña,§ M. Carmen López Velasco,‡ Ricela E. Sellek,* M. Rosario Jiménez Chillarón,‡ Luisa P. Sánchez Serrano,* and J. Fernando Martínez Navarro***

*Instituto de Salud Carlos III, Majadahonda, Madrid, Spain; †Public Health Department, Alcalá de Henares, Madrid, Spain; ‡Cuenca Public Health Department, Cuenca, Spain; and §Ministry of Agriculture and Fisheries, Algete, Madrid, Spain

In 1997, an outbreak of human tularemia associated with hare-hunting in central Spain affected 585 patients. We describe the identification of *Francisella tularensis* biovar palaearctica in a second outbreak of ulceroglandular tularemia associated with crayfish (*Procambarus clarkii*) fishing in a contaminated freshwater stream distant from the hare-associated outbreak. The second outbreak occurred 1 year after the first.

Human tularemia is a rare but highly virulent bacterial zoonosis with endemic foci in the Northern Hemisphere (1). Its clinical manifestations depend on the route of infection. The ulceroglandular form, the most common, occurs after handling contaminated sources. Ingestion of contaminated food or water can cause an oropharyngeal form. Pulmonary, typhoidal, glandular, and ocular forms are less frequent. The disease occurs in outbreaks, usually associated with direct contact with infected game or contaminated water, or in a seasonal pattern in arthropodborne tularemia (2). *Francisella tularensis* is the causative agent (3). Two main biovars are included in this species: the most virulent (Jellison type A or *F. tularensis* biovar tularensis), described mainly in North America and recently reported from central Europe (4), and a less virulent (Jellison type B or *F. tularensis* biovar palaearctica), mainly found in Eurasia and to a lesser extent in North America (5). The current third biovar, type C (*F. tularensis* biovar novicida) (6), was formerly considered one of the three species of the genus (3). Types A and B are related to human disease as the cause of severe and mild tularemia, respectively. Type C has been isolated from water and is an infrequent cause of disease in humans (6).

This microorganism is perpetuated in nature in an enzootic cycle involving wild mammals (mainly rodents and lagomorphs) and invertebrates (ixodid ticks, mosquitoes, tabanids, and other bloodsucking arthropods). The reservoir has not been clearly assessed, although the disease can be passed in nature by tick bite (2) and both transtadial and transovarial transmission have been described in ticks (7). While *F. tularensis* can survive for months in cold water, it is adversely affected by direct sunlight and hot temperatures.

Several enzootic cycles have been described in the Old World. Direct contact with infected hares accounts for most human cases in Western and Central Europe and the former Soviet Union, where water-related cases have also been described. Mosquitoes are the main vector for infection of humans and hares in northern Europe, and tick- and airborne cases have also been reported (2).

This disease is uncommon in southern Europe, but cases have occurred in Italy and France (8-10). In Spain, apart from the retrospective identification in 1999 of one ulceroglandular case acquired in 1996 (11), the first human cases were identified in 1997 (12,13), when a hare-associated outbreak affected 585 patients (14; and references thereafter in same issue). Since then, a few sporadic cases have been diagnosed in the same area.

We describe the identification of *F. tularensis* biovar palaearctica in a second outbreak of ulceroglandular tularemia associated with crayfish (*Procambarus clarkii*) fishing in a contaminated freshwater stream distant from the hare-associated outbreak.

### Methods

### Subjects

A confirmed patient was defined as a person with compatible signs and symptoms (ulcerated lesions in the hands and regional lymphadenopathies with or without fever and general discomfort) and an accompanying positive laboratory result (seroconversion or single antibody titer ≥1:128 to *F. tularensis* as measured by microagglutination or a positive polymerase chain reaction [PCR] result).

### Epidemiologic Study

Several visits were made to the epidemic site. Fishing areas were identified by photographs that were subsequently shown to patients and controls. Interviews were conducted with local persons to obtain relevant information and with experts in red swamp crayfish ecology. Mean monthly dam water levels for 1998 were obtained.

An active search was made for compatible cases and for persons reporting possible contact with any potential reservoir of the disease. Health-care centers were alerted. We

Address for correspondence: Pedro Anda, Servicio de Bacteriología, Centro Nacional de Microbiología-Instituto de Salud Carlos III, 28220-Majadahonda, Madrid, Spain; fax: 3491-509-7966; e-mail: panda@isciii.es searched reports of emergency visits (for January 1 to August 31, 1998) and discharges (for January 1 to September 30, 1998) at the provincial hospital for patients who met the case definition or had tularemia-compatible symptoms.

Patients were interviewed to evaluate the probability of any contact with other tularemia reservoirs, pinpoint the fishing site, and define risk factors linked to crayfish fishing. We selected 20 controls who did not meet the case definition, had been in contact with crayfish caught on the same dates and in the same places, and generally resided in the same towns as patients. All patients and controls were interviewed from October 8 through December 7, 1998.

The crude odds ratio (OR), its 95% confidence intervals (CI), as well as adjusted OR, were determined by logistic regression.

### Bacterial Strains and Culture

The live vaccine strain of *F. tularensis* type B (NCTC 10857) was used for serologic diagnosis and as a positive control for PCR. The strain was grown in modified Thayer-Martin (Difco Laboratories, Detroit, MI) chocolate, supplemented with Isovitalex (10 mg/L; BBL Microbiology Systems, Cockeysville, MD) and 1% L-Cysteine (Sigma-Aldrich Co., Alcobendas, Madrid, Spain) at 37°C in 5% $CO_2$ (3,13).

### Environmental Samples

Eight water samples were collected at several points on the river where all patients had fished. Three water samples from a sewage plant located 15 km upstream were also collected. Water sampling was repeated 1 month later. Three crayfish captured by one patient had been frozen and were available for study (batch A). Twenty more crayfish were collected from two stretches of the river where the outbreak had started (batch B). Sixteen hares (*Lepus* spp.) (12 alive; 4 dead), 7 common shrews (*Sorex araneus*), 26 field mice (*Apodemus sylvaticus*), 2 gudgeons (*Gobio gobio*), 3 rats (*Rattus rattus*), 1 badger (*Meles meles*), and 1 common vole (*Microtus arvalis*), as well as sera from 60 sheep from the epidemic area were also studied. Wild mammals were euthanized in a $CO_2$ chamber and both liver and spleen cultured (13).

Intestines of batch A crayfish were not available for study as they were removed in situ and arrived at the laboratory after being extensively washed in tap water. Consequently, branchial tissue from three crayfish, one stomach, two gonads, and one hepatopancreas were aseptically collected and thinly ground with a scalpel. The carcasses were immersed in autoclaved distilled $H_2O$, shaken at 33°C for 30 min, centrifuged at 4,000 x *g* for 15 min at 4ºC, and the pellet (named wash) resuspended in 100 µL of autoclaved distilled $H_2O$. The rest of the process was as described above. For batch B crayfish, intestines were also dissected and studied. All fractions were plated in the same media as above.

Water samples (10 mL) were concentrated by centrifugation at 8,000 x *g* for 15 min and the pellet resuspended in 1 mL of the original sample and subjected to acidic shock, as described for *Legionella pneumophila* (15). Briefly, 0.1 mL of each concentrated sample was mixed with 0.9 mL of buffer HCl/KCl pH 2.2, and after an incubation of 5 min at room temperature, the samples were plated. This method was based on the assumption that the survival capacities of these microorganisms in the intracellular environment could account for resistance to acidic solutions and reduce the level of contaminants. Individual colonies that looked similar to *F. tularensis* live vaccine strains were pooled in groups of 10 in 100 µL of autoclaved distilled $H_2O$, carefully mixed, and half the volume was reserved for PCR. The rest of these samples were kept at 4°C.

### Microaglutination Test

Agglutinating antibodies against whole *F. tularensis* live vaccine strain were determined as described (16,17). Titers $\geq$1:64 were considered positive.

### PCR Detection of *F. tularensis*

A lymph node aspirate from one of the patients was subjected to guanidine thyocianate lysis for DNA extraction as described (18). All reagents were purchased from Sigma-Aldrich, except for glycogen and proteinase K (Boehringer Mannheim, Roche Molecular Systems, Inc., Branchburg, NJ).

For the water samples, 1 mL of each was centrifuged at 15,000 x *g* for 10 min, and the pellet was resuspended in 100µL of the same sample and clarified by a 5-min centrifugation at 3,000 x *g* to eliminate insoluble material. The supernatant was then collected, lysed as above, and DNA precipitated and resuspended with 10 µL of autoclaved distilled $H_2O$.

For the crayfish, all fractions studied by culture were also digested as above and subjected to PCR by using the 16S ribosomal DNA-based universal F1-R13 and *Francisella*-specific F5-F11 sets of primers described by Forsman et al. (19) in a nested PCR (Table 1).

The F5-F11 amplicons obtained from water, lymph node aspirates, and crayfish samples were sequenced by PCR with

Table 1. Polymerase chain reaction results of crayfish and water samples tested for *Francisella tularensis*[a]

| Crayfish, Batch A | | | | | Crayfish, Batch B | | | | | | Water SP | | | Water R | Patient |
|---|---|---|---|---|---|---|---|---|---|---|---|---|---|---|---|
| B | S | H | G | W | B | S | H | G | W | I | 1 | 2 | 3 | 1-8 | LNA |
| - | + | + | - | - | - | - | - | - | - | - | + | - | 3 | - | + |

Batch A = crayfish collected from a patient's house; Batch B = crayfish collected from the river; SP = sewage plant; R = river; LNA = lymph node aspirate; B = branchia; S = stomach; G = gonads; H = hepatopancreas; W = washed pellet; I = intestines.

[a]Several annealing temperatures were tested, from 60°C to 68°C for the 25 cycles of the first round. The second round consisted of 45 cycles with an annealing temperature of 60°C. The rest of the parameters were as described (21). All reagents were from Perkin Elmer (Foster City, CA); the cycling was done in a PCT-100 thermocycler (MJ Research Inc., Watertown, MA). The PCR products, with a size of 1550 bp and 950 bp for the first and second rounds of amplification, respectively, were visualized in 1% low-melt agarose gels (Pronadisa, Alcobendas, Madrid, Spain) stained with ethidium bromide (Sigma-Aldrich). Cross-contamination was avoided by using standard methods. Half the samples studied were kept unprocessed and frozen in a different area. A positive result was confirmed by processing the rest of the sample. To assess the sensitivity of our system, 10 µL of serial twofold dilutions of an aqueous suspension of bacteria, ranging from $10^4$ CFU to $10^2$ mL was added to 1-mL volumes of distilled water. These samples were subjected to DNA extraction and PCR as above. Negative controls (autoclaved distilled $H_2O$) were included in all extractions at a ratio of a negative control for each five samples. The specificity was checked against DNA from *Salmonella* Typhimurium, *Escherichia coli, Legionella pneumophila, Yersinia enterocolitica,* and *Proteus vulgaris*.

the BigDye Terminator Cycle Sequencing kit on an ABI 377 automated DNA sequencer (Perkin-Elmer Corp., Foster City, CA) following the manufacturer's instructions. Sequence homology was searched by using the BLAST 2.0 queueing system (20).

### Results

### Patients and Serology

Nineteen patients who fulfilled laboratory criteria for tularemia were identified. All had had contact with river-caught crayfish in the period July 13-31. Overall, 11 (58%) required hospitalization, with a mean stay of 9.4 days (Table 2). Symptoms included adenopathies on the elbow, armpit, or both (100%); cutaneous lesions (89%); fever (80%); discomfort at the injury site (42%); vomiting (32%); diarrhea (5%); and general symptoms (84%) such as headache, arthralgia-myalgia, malaise, anorexia, dysuria, and bad taste in the mouth.

All cutaneous lesions were on hands, generally the fingers; no specific finger or hand predominated. In six cases, ulcers were present. The remaining patients reported inflammation of the finger or phalanx to which they had received the crayfish-related injury. All ulcers were at the injury site.

Forty-one serial serum samples from all the patients were collected (Table 2). All 19 patients reached *F. tularensis* specific microagglutination titers ≥1:256. Increases in serum antibody titers were observed in five cases (Table 2). For the remaining patients, high antibody titers in the presence of compatible clinical symptoms and full recovery after antibiotic treatment were interpreted as confirmatory criteria.

Serum samples from wild animals and sheep were negative.

Table 2. Serologic results and date of onset of tularemia, 19 patients

| Patient no. | Days since onset | MA titer | Sex | Age (years) | Exposure date/ onset date | Hospitalization/ other laboratory tests |
|---|---|---|---|---|---|---|
| 1 | 40 | 4,096 | F | 71 | 7-14/7-16 | 15 d |
| | 69 | 4,096 | | | | |
| 2 | 38 | 2,048 | F | 63 | 7-13/7-18 | 11 d |
| | 69 | 2,048 | | | | |
| 3 | 52 | 256 | M | 72 | 7-15/7-17 | NO |
| | 69 | 1,024 | | | (+) PCR | |
| 4 | 44 | 2,048 | F | 71 | 7-16/7-18 | 15 d |
| | 66 | 2,048 | | | | |
| 5 | 38 | 512 | F | 62 | 7-14/7-18 | 14 d |
| | 58 | 512 | | | | |
| 6 | 38 | 256 | F | 53 | 7-14/7-18 | NO |
| | 54 | 256 | | | | |
| | 82 | 256 | | | | |
| 7 | 36 | 512 | M | 39 | 7-17/7-20 | NO |
| | 53 | 512 | | | | |
| 8 | 22 | 16 | M | 54 | 7-19/7-21 | 7 d |
| | 42 | 256 | | | | |
| | 64 | 256 | | | | |
| 9 | 17 | 2,048 | M | 38 | 7-15,22/7-24 | NO |
| | 94 | | | | | |
| 10 | 74 | 512 | F | 60 | 7-20/7-23 | NO |
| | 95 | 512 | | | | |
| 11 | 47 | 512 | M | 51 | 7-22/7-24 | 5 d |
| | 52 | 2,048 | | | | |
| 12 | 47 | 2,048 | M | Unknown | 7-22/7-25 | 9 d |
| | 61 | 2,048 | | | | |
| 13 | 30 | 1,024 | M | 48 | 7-19/7-26 | 7 d |
| | 50 | 1,024 | | | | |
| 14 | Unknown | 256 | F | 65 | 7-15/Unknown | NO |
| 15 | 16 | 16 | M | 75 | 7-25/7-27 | 9 d |
| | 52 | 256 | | | | |
| | 73 | 256 | | | | |
| 16 | 42 | 256 | F | 60 | 7-27/7-30 | NO |
| | 55 | 256 | | | | |
| | 70 | 256 | | | | |
| 17 | 18 | 2,048 | M | 49 | 7-30/8-2 | 6 d |
| | 38 | 2,048 | | | | |
| 18 | 22 | 512 | M | 67 | 7-30/8-3 | 6 d |
| | 53 | 512 | | | | |
| 19 | 103 | 8,192 | M | 65 | 7-26/8-5 | NO |
| | 178 | 2,048 | | | | |

MA = microagglutination; d = day; PCR = polymerase chain reaction; NO = no hospitalization required.

### Case Survey and Risk Factors

Patients included 11 men and 8 women, mean age 59.1 years (range 38-75); the occupations of housewife (7 patients) and pensioner (5) predominated. None had professions entailing risk practices for tularemia. Most had been catching and cleaning crayfish for many years.

Cases were concentrated (by date of symptom onset) within a 3-week period (July 16-August 5; Table 2 and Figure 1); the greatest concentration of cases occurred during July 17-26. All patients had been in contact with crayfish caught during July 13-30. Fishing had been officially permitted on June 1 but was prohibited on August 6 because of the outbreak. The estimated incubation period was 3.5 days (range 1-8 days).

Patients resided in six towns situated around the Mayor River fishing area. Two patients had been in contact with other tularemia reservoirs (hares). No insect bites were reported for any patients.

All crayfish caught were of the red swamp species (*P. clarkii*). The section of the river authorized for fishing is 14 km, from the town of Huete to the beginning of the Buendía

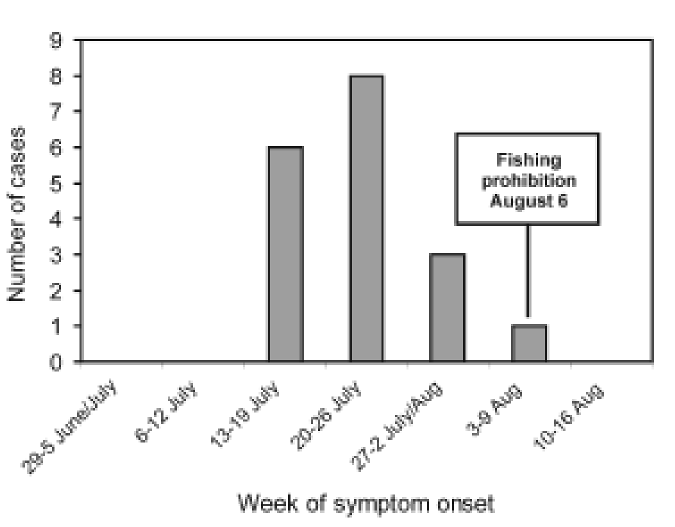

Figure 1. Epidemic curve of tularemia cases, central Spain, by week. One case had unknown symptom-onset date.

Dam into which the river flows. There are designated hunting (including hare-hunting) areas on both river banks. Patients had been fishing a ±5-km stretch, particularly the area at the neck of the dam, where access to the river was simplified by the proximity of the road and two footbridges (Figure 2). This terrain is alluvial, so crayfish can easily dig their underground burrows (22); it is prone to periodic variations in the water level and marked by reeds. The abundance of crayfish favored fishing by hand (an outlawed practice) rather than with a crayfish net.

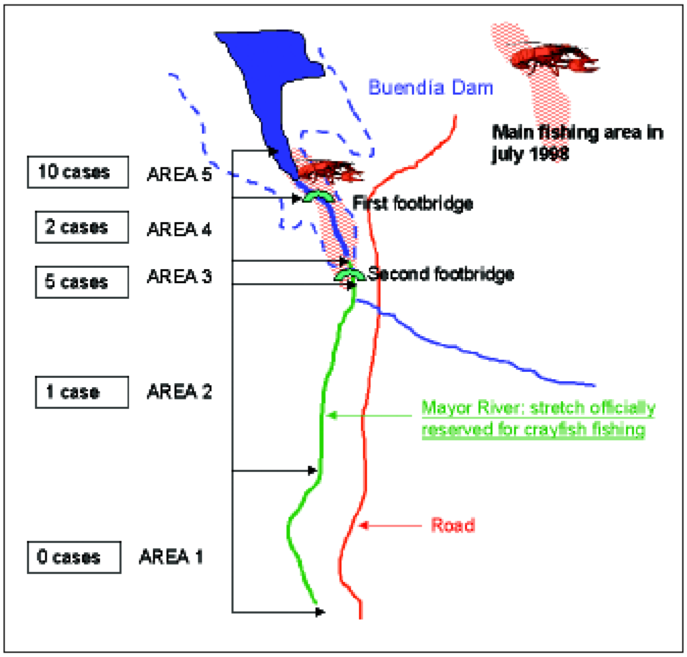

Figure 2. Fishing areas where crayfish handled by patients were caught. (Data were unavailable for one patient.)

Our case survey showed that 113 patients had caught crayfish; at least 9 of these patients had also cleaned them. Six patients had cleaned crayfish but had neither caught them nor been in the fishing area; five of these six had cleaned crayfish the day after the catch, and one had done so 5 days afterwards. In all, 15 patients had cleaned crayfish, and at least 17 of the total 19 had also eaten crayfish. Cooking methods used were all low risk.

At least 17 patients had incurred crayfish-related scratches, cuts, and abrasions while fishing, emptying the net, or cleaning the catch; 8 had only one injury. One patient, who had been injured when fishing and cleaning, also grazed against a riverside reed; the cutaneous lesion subsequently appeared at the site of the scratch.

Water used for washing crayfish was from the residential drinking water supply; chlorine levels were checked and shown to be acceptable. In 9 (75%) of 12 respondents who cleaned crayfish, the washing method consisted of leaving the crayfish to soak and changing the water several times. During this operation, a considerable quantity of mud and dirt was released, which the cleaner touched. In the remaining three cases, crayfish were rinsed in running tap water. These two washing operations were performed by 58% (7/12) of patients before they gutted the crayfish. All but one of the 11 respondents reported washing their hands after handling the crayfish, but 5 of those 10 used no soap or disinfectant.

### Case-Control Study

Demographic information on patients was obtained (Table 3). Although there was a higher proportion of women and a lower proportion of crayfish catchers among controls, these differences were not statistically significant.

The most important result (Table 4) was that those persons who were injured during the handling (catching or cleaning) of crayfish had a significantly higher risk for tularemia (94% of exposed subjects among cases versus 30% among controls; OR = 39.7; 95% CI 4.3-369.7). Furthermore, once data were reciprocally adjusted for the catching- and cleaning-related injury variables, merely incurring an injury (regardless of whether it was received while catching crayfish [OR adjusted for cleaning-related-injury = 29.1; 95% CI 2.6-330.9] or cleaning crayfish [OR adjusted for catching-related-injury = 38.8; 95% CI 3.5-427.6]) led to a statistically significant rise in the risk for tularemia.

### Environmental Study

Local environmental variations detected over the previous 2 years were higher rainfall, with correspondingly higher river levels; abundant crayfish; and increased murkiness of the river water. In July 1998, release of water through the sluice gates led to an estimated 1.8-m drop in the overall dam level, leading in turn to a 200- to 400-m narrowing in the shoreline at the neck of the dam, i.e., the fishing area (Figure 3). This coincided with a mass movement of crayfish to the surface.

### Culture and PCR

Attempts to culture the organism from water, patients, crayfish, and wild animals were unsuccessful. A positive PCR result was obtained from one of the pools of 10 individual colonies taken from the culture of a water sample from the sewage plant, but we were unable to recover any *F. tularensis* in culture from the rest of the same sample kept at 4°C.

The PCR conditions best adapted to our system were an annealing temperature of 62°C for the first round of cycles and 60°C for the second. These conditions allowed us to

Table 3. Comparison of characteristics, tularemia cases versus controls

| Variable | Cases [a] | Controls | p |
|---|---|---|---|
| Age: mean (range) in years | 59.1 (38-75) | 56.2 (25-100) | 0.7 |
| Sex: | | | |
| Women | 42% ( 8/19) | 55% (11/20) | 0.4 |
| Men | 58% (11/19) | 45% (9/20) | |
| Town of residence: | | | |
| No.1 | 3 | 3 | |
| No.2 | 6 | 7 | |
| No.3 | 4 | 4 | |
| No.4 | 4 | 3 | |
| No.5 | 1 | 2 | |
| No.6 | 1 | 0 | |
| No.7 | 0 | 1 | |
| Type of contact with crayfish: | | | |
| Catching | 68% (13/19) | 82% (15/18) | 0.6 |
| Cleaning | 94% (17/18) | 85% (17/20) | 0.9 |
| Eating | 60% (12/20) | 95% (19/20) | 0.9 |

[a]The denominator indicates the number of persons who answered each question.

Table 4. Results of case-control study of tularemia in crayfish

| | Prevalence of exposure[a] in | | | | | |
|---|---|---|---|---|---|---|
| Risk factors considered | Cases | Controls | Crude OR | 95% CI | Adjusted OR | 95% CI |
| Injury on handling crayfish | 17/18 | 6/20 | 39.7 | 4.3-369.7 | | |
| Injury on catching[b] crayfish | 8/17 | 3/20 | 5.0 | 1.1-23.8 | 29.1[c] | 2.6-330.9 |
| Injury on cleaning crayfish | 10/17 | 3/20 | 8.1 | 1.7-38.6 | 38.8[d] | 3.5-427.6 |
| Not washing crayfish before cleaning | 5/12 | 4/17 | 2.3 | 0.4-15.7 | | |
| Washing crayfish by soaking | 9/12 | 10/17 | 2.1 | 0.4-10.7 | | |
| Washing hands with soap after cleaning | 5/11 | 11/15 | 0.3 | 0.06-1.6 | | |

[a] Denominator indicates number of persons who answered each question.
[b] Injury on catching = injury while fishing or emptying net.
[c] Adjusted for cleaning-related injury.
[d] Adjusted for catching-related injury.
OR = odds ratio; 95% CI = 95% confidence interval.

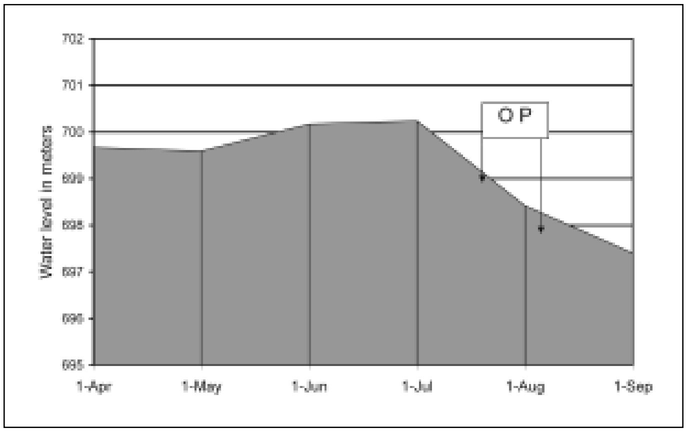

Figure 3. Buendía Dam water levels, April-September 1998. OP = outbreak period.

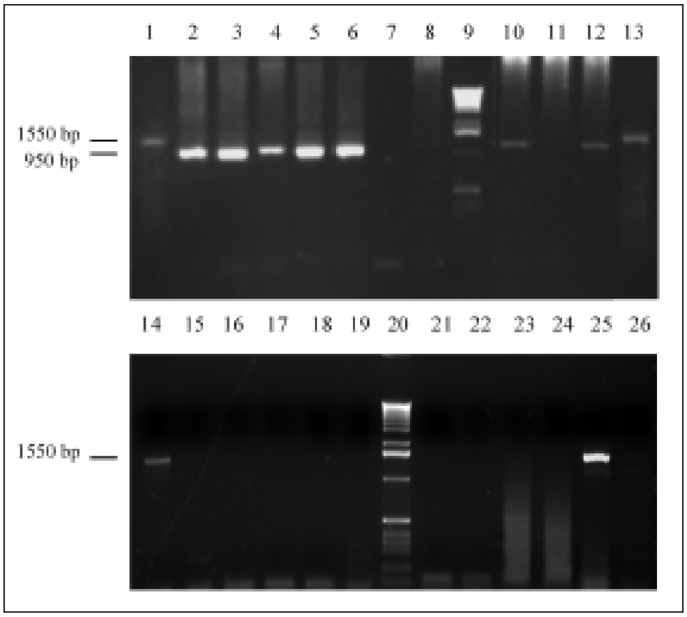

Figure 4. Polymerase chain reaction results in selected samples from water, crayfish, patients, and controls. Lanes 1 and 2: positive control (first [1550-bp] and second [950-bp] rounds of amplification); lanes 3 and 4: water samples 1 and 3, respectively, from the sewage plant, second round; lanes 5 and 6: stomach and hepatopancreas, respectively, from batch A of crayfish, second round; lane 7: negative control; lanes 8 and 10: control of amplification of 1 CFU for the first and second rounds of amplification, respectively; lanes 11 and 12: human lymph node aspirate, first and second amplifications, respectively; lanes 13 and 14: positive control, first round; lanes 15-19, 21, and 22: water samples from the river; lanes 23 and 24: stomach and hepatopancreas, respectively, from batch B of crayfish; lanes 25 and 26: pooled DNA from *Salmonella* Typhimurium, *Escherichia coli, Legionella pneumophila, Yersinia enterocolitica,* and *Proteus vulgaris* (first and second rounds of amplification, respectively); lanes 9 and 20, 1-kb DNA ladder size standards.

amplify DNA from 1 CFU of spiked distilled water, which yielded a faint band only in the second amplification run (Figure 4), as did the lymph node aspirate from a patient (Figure 4 and Table 1).

Two of three samples from the sewage plant yielded amplicons, although those samples were negative in culture. Stomach and hepatopancreas samples from the crayfish specimens collected at a patient's house were positive, and branchial tissue, gonad, and the washed pellet were negative. All water samples collected from different parts of the river were negative. PCR results of three additional water samples collected 1 month later from the same locations yielded the same results. Intestinal, stomach, and hepatopancreas samples from crayfish collected from the river after the outbreak started were negative (Figure 4, Table 1).

### Sequencing of 16S rDNA

An identical fragment of the 16S rDNA gene (nucleotide position 145-1290 as for *Escherichia coli*) was sequenced from the water, crayfish, and lymph node aspirate amplicons and analyzed. The homology in this region between the lymph node aspirate amplicon and the published sequences for *F. tularensis* tularensis, *F. tularensis* palaearctica, *F. novicida, F. phylomiragia, Salmonella* Typhimurium*, Legionella pneumophila,* and *E. coli* was determined (Table 5). The lymph node aspirate sequence shared 100% of homology with the sequence L26086 from *F. tularensis* palaearctica (99.2% with *F. tularensis* tularensis and *F. novicida*, and 97.7% with *F. philomiragia*). The homology with *S.* Typhimurium*, E. coli,* and *L. pneumophila* was 81.0%, 81.1%, and 80.8%, respectively. When a shorter fragment was analyzed, between nucleotide positions 1113 and 1188, the sequence of the PCR products obtained had 100% homology with *F. tularensis* palaearctica, 98.6% with *F. tularensis* tularensis, 99.3% with *F. novicida*, and 98.9% with *F. philomiragia* (data not shown). In this region, we found 15 of 75 nt positions specific for *Francisella* spp., which implies a divergence of 20% compared with *S.* Typhimurium*, L. pneumophila*, and *E. coli* (Figure 5A). All sequences generated shared the nucleotide signature that differentiates *F. tularensis* palaearctica from other

Table 5. 16S rRNA gene sequence similarity matrix (from position 145 to 1290 as for *Escherichia coli*)

| | LNA | Ftp | Ftt | Fn | Fph | Lp | Ec | St |
|---|---|---|---|---|---|---|---|---|
| LNA | 100 | | | | | | | |
| Ftp | 100 | 100 | | | | | | |
| Ftt | 99.2 | 99.2 | 100 | | | | | |
| Fn | 99.2 | 99.2 | 98.6 | 100 | | | | |
| Fph | 97.7 | 97.7 | 97.2 | 100 | | | | |
| Lp | 80.8 | 80.8 | 80.3 | 80.2 | 80.8 | 100 | | |
| Ec | 81.1 | 81.1 | 80.5 | 80.5 | 80.3 | 80 | 100 | |
| St | 81 | 81 | 80.8 | 80.4 | 80 | 79.7 | 95.9 | 100 |

LNA: human lymph node aspirate amplicon, this study (GenBank Accession No. AF227314); Ftp: *F. tularensis* palaearctica (L26086); Ftt: *Francisella tularensis* tularensis (Z21932); Fn: *F. novicida* (L26084); Fph: *F. philomiragia* (Z21933); Lp: *Legionella pneumophila*; (M36023); Ec: *Escherichia coli* (AE000406); St: *Salmonella* Typhimurium (X80681).

members of the genus (an A at nucleotide position 1153, as described for *F. tularensis* palaearctica, compared with a G, as described for *F. tularensis* type A, *F. novicida,* and *F. phylomiragia)* (23) (Figure 5A).

### Conclusion

We describe the identification by PCR of *F. tularensis* biovar palaearctica as the agent of a waterborne outbreak of tularemia. The fragments of the 16S rRNA gene sequenced were specific for *Francisella* spp. The homology of these fragments with the members of the *Francisella* genus and the nucleotide signature at position 1153 confirmed that this organism caused this outbreak. The results obtained are compatible with a transient contamination of the river and crayfish. A sewage plant, which intermittently pours water into the river 15 km upstream, could be the source of the organisms, as the samples taken 1 month after the outbreak began remained positive by PCR. Alternatively, crayfish could have acquired the organism during a previous contamination of the river and maintained the bacteria in their internal organs, as water samples taken from the river a few days after the outbreak started were negative by PCR. That the crayfish captured 4 weeks after the outbreak started were negative by PCR and that the only positive ones were those recovered from the house of a patient indicated that contamination was transient and that the organism cleared from the digestive tract of the crayfish.

Waterborne human tularemia is usually oropharyngeal. We have described an unusual waterborne outbreak of ulceroglandular tularemia. As the patients' skin lesions were granuloma-like, a *Mycobacterium marinum* infection (24) was at first suspected, thus delaying extraction of the first serum samples. In spite of this, we were able to identify the cause of the illness by serology and PCR.

Data indicate that the *F. tularensis* complex is highly variable in Eurasia (4,8,9,25-27). Whether this variability could account for the microorganism's ability to survive and multiply in different ecosystems and lead to different forms of transmission to humans deserves further study.

The presence of *F. tularensis*, both in the sewage-plant water and in the river crayfish, along with the association between the disease and hand injuries incurred on coming into contact with river water and mud at the fishing site or during crayfish cleaning, led us to conclude that the river is contaminated with *F. tularensis*. The beginning of the official open fishing season and the emergence of large number of crayfish to the surface (28) brought on by the drop in river levels in July enhanced the possibility of contact with the crayfish and thus the outbreak.

The temporal-spatial features of the outbreak suggest that contamination of the river must have been limited in time and to a single stretch of the river. Favoring this hypothesis is the fact that bacteria were not found in specimens of potential animal reservoirs in the area.

Evidently, the crayfish played the role of mechanical transmission agents of disease to humans, through contact

Figure 5. A) Sequence alignment of a 75-nt fragment of the 16S rRNA gene of different bacterial species (nucleotide position 1113 to 1188 as for *Escherichia coli*). The signature nucleotide (nt 1153), which allows differentiation between *F. tularensis* palaearctica and the other members of the *Francisella* genus, is set in bold face. B) Sequence comparison of the regions corresponding to F11- and F5-specific *Francisella tularensis* primers between *F. tularensis* and other bacterial species. Asterisks depict *Francisella* spp.-specific nucleotides. Sequences generated in this study were deposited in GenBank with accession numbers of AF227312 for the amplicon from water, AF227313 for the fragment amplified from crayfish stomach, and AF227314 for the human lymph node aspirate. Sequences generated in this study were compared with those of *Salmonella* Typhimurium (X80681), *Escherichia coli* (AE000406), *Legionella pneumophila* (M36023), *F. tularensis* biovar tularensis (Z21932), *F. tularensis* biovar palaearctica (L26086), *F. novicida* (L26084), and *F. philomiragia* (L26085).

with carapace-contaminated mud and water; skin injuries acted as the portals of entry for infection. The presence of *F. tularensis* in the crayfish stomach and hepatopancreas, coupled with this type of crayfish's known capacity for bioaccumulation of toxins, poses the possibility of transmission through contact with the intestinal contents during the gutting process.

Commercial farming of this crayfish species is expanding internationally because of its high profitability (28). Thus, the potential role of this species as a vector for tularemia needs further study.

*F. tularensis* has been classified as one of the microorganisms that could be used as a biological warfare agent (29-31). The waterborne route for infection that we have described here supports the possibility that intentional contamination of water could be the source of a rare bacterial disease in the future. Thus, it is important to consider these factors at the clinical and public health levels.

### Acknowledgments

The authors thank E. Chaparro and I. Rodríguez for their technical help; May C. Chu for help in setting up the serologic test; Marta Zimmerman Verdejo for help with statistical analysis and manuscript review; José Luis Múzquiz for analyses of animal specimens and water samples; José María Martínez and Fernando Alonso for information on the ecology of the red swamp crayfish; local health-care staff, including all veterinary surgeons, pharmacists, and medical practitioners in the towns with patients, and especially Lucinda Velázquez, who first suspected the outbreak; Cuenca's Virgen de la Luz Hospital; local and regional authorities, including Margarita Abel Pareja, Gonzalo Gutierrez Avila, and the Huete Town Council, in particular, for their support. Finally, we thank all patients, their families, and those who agreed to act as controls.

This work was partially financed by Fondo de Investigación Sanitaria (FIS) 98/026-01 and NATO CRG 950380. Raquel Escudero was financed by a fellowship from the Fondo de Investigación Sanitaria. Ricela Sellek was financed by a "Beca de Iniciación" of the Instituto de Salud Carlos III.

Dr. Anda is a microbiologist in the National Center for Microbiology-Instituto de Salud Carlos III, Majadahonda, Madrid, Spain. He works in the diagnosis of bacterial zoonoses, and his research interests focus on the pathogenesis of human borrelioses.

### References

1. Hopla CE. The ecology of tularemia. Adv Vet Sci Comp Med 1974;18:25-53.
2. Mörner T. The ecology of tularemia. Rev Sci Tech Off Int Epiz 1992;11:1123-30.
3. Eigelsbach HT, McGann VG. Gram-negative aerobic cocci. In: Krieg NR, Holt JG, editors. Bergey's manual of systematic bacteriology. 1st ed (vol 1). Baltimore: Williams & Wilkins Co; 1984. p. 394-9.
4. Gurycova D. First description of *Francisella tularensis* subsp. tularensis in Europe. Eur J Epidemiol 1998;14:797-802.
5. Olsufjev NG, Emelyanova OS, Dunayeva TN. Comparative study of strains of *Bacterium tularense*. II. Evaluation of criteria of virulence of *Bacterium tularense* in the old and the new world and their taxonomy. J Hyg Epidemiol Microbiol Immunobiol 1959;3:138-49.
6. Hollis DG, Weaver RE, Steigerwalt AG, Wenger JD, Moss CW, Brenner DJ. *Francisella phylomiragia* com. nov. (formerly *Yersinia philomiragia*) and *Francisella tularensis* biogroup novicida (formerly *Francisella novicida*) associated with human disease. J Clin Microbiol 1989;27:1601-18.
7. Hubálek Z, Halouzka J. Mosquitoes (Diptera: Culicidae), in contrast to ticks (Acari: Ixodidae), do not carry *Francisella tularensis* in a natural focus of tularemia in the Czech Republic. J Med Entomol 1997;34:660-3.
8. Mignani E, Palmieri F, Fontana M, Marigo S. Italian epidemic of waterborne tularaemia. Lancet (letter) 1988;2:1423.
9. Greco D, Allegrini G, Tizzi T, Ninu E, Lamanna A, Luzi S. A waterborne tularemia outbreak. Eur J Epidemiol 1987;3:35-8.
10. Le Coustumier AI, Le Coustumier A, Artois M, Audurier A, Barrat J, Couetdic G, et al. Epidémiologie de la tularémie en France: modes de transmission inhabituels, recrudescence en 1993 de la tularémie humaine. Bulletin Épidémiologique Hebdomadaire 1994;42:195-7.
11. Campos A, Merino FJ, Nebreda T, García-Peña FJ, Sanz-Moncasí P. Diagnóstico retrospectivo del primer caso de tularemia asociado a contacto con liebre en España. Enf Infecc Microbiol Clin 1999;17:417-8.
12. Instituto de Salud Carlos III. Centro Nacional de Epidemiología. Brote de tularemia en Castilla-León. Boletín Epidemiológico Semanal 1997;5:249-52.
13. García-Peña FJ, Suárez Mayoral P, Cogolludo Cogolludo C, Arriola Garrote C, Anadón Navarro E. Brote de tularemia en la comunidad autónoma de Castilla-León. Primer aislamiento en España de *Francisella tularensis*. Med Vet 1998;15:418-23.
14. Eiros Bouza JM, Rodríguez-Torres A. Tularemia. Rev Clin Esp 1998;198:785-8.
15. Mahony MCO, Stanwell-Smith RE, Tillet HE, Harper D, Hutchison JGP, Farrell ID, et al. The Stafford outbreak of Legionnaire's disease. Epidemiol Infect 1990;104:361-80.
16. Massey ED, Mangiafico JA. Microaagglutination test for detecting and measuring serum agglutinins of *Francisella tularensis*. Appl Microbiol 1974;27:25-7.
17. Brown SL, McKinney FT, Klein GC, Jones WL. Evaluation of a Safranin-O stained antigen microagglutination test for *Francisella tularensis* antibodies. J Clin Microbiol 1980;11:146-8.
18. Casas I, Powell L, Klapper PE, Cleator GM. New method for the extraction of viral RNA and DNA from cerebrospinal fluid for use in the polymerase chain reaction assay. J Virol Meth 1995;53:25-36.
19. Forsman M, Sandström G, Sjöstedt A. Analysis of 16S ribosonal DNA sequences of *Francisella* strains and utilization for determination of the phylogeny of the genus and for identification of strains by PCR. Int J Sys Bacteriol 1994;44:38-46.
20. Altschul SF, Madden TL, Schäffer AA, Zhang J, Zhang Z, Miller W, et al. Gapped BLAST and PSI-BLAST: a new generation of protein database search programs. Nucleic Acids Res 1997;25:3389-402.
21. Forsman M, Nyrén A, Sjöstedt A, Sjökbvist L, Sandström G. Identification of *Francisella tularensis* in natural water samples by PCR. FEMS Microbiol Ecol 1995;16:83-92.
22. Hasiotis S. Notes on the burrow morphologies and nesting behaviours of adults and juveniles of *Procambarus clarkii* and *Procambarus acutus acutus* (*Decapoda: Cambaridae*). Freshwater Crayfish 1995;8:623-34.
23. Forsman M, Sandström G, Jaurin B. Identification of Francisella strains and discrimination of Type A and Type B strains of *F. tularensis* by 16S rRNA analysis. Appl Environ Microbiol 1990;56:949-55.
24. Cramer HJ, Hottenrott G. Tuberculosis resembling skin disease caused by *Mycobacterium marinum* (so-called swimming pool granuloma). Dtsch Gesundheitsw 1967;22:702-5.
25. Greco D, Ninu E. A family outbreak of tularemia. Eur J Epidemiol 1985;1:232-3.
26. Mörner T, Mattsson R, Forsman M, Johansson K-E, Sandström G. Identification and classification of different isolates of *Francisella tularensis*. J Vet Med 1993;40:613-20.
27. Sandström G, Sjöstedt A, Forsman M, Pavlovich NV, Mishankin BN. Characterization and classification of strains of *Francisella tularensis* isolated in the central Asian focus of the Soviet Union and Japan. J Clin Microbiol 1992;30:172-5.

28. Huner JV. *Procambarus* in North America and elsewhere. In: Holdich DM, Lowery RS, editors. Freshwater crayfish: Biology, management and exploitation. London: Croom Helm Press; 1988. p. 239-61.
29. McGovern TW, Christopher GW, Eitzen EM. Cutaneous manifestations of biological warfare and related threat agents. Arch Dermatol 1999;135:311-22.
30. Henderson DA. The looming threat of bioterrorism. Science 1999;26:1279-82.
31. Kortepeter MG, Parker GW. Potential biological weapons threats. Emerg Infect Dis 1999;5:523-7.
